# Do oncologists prefer subspecialty radiology reports? A quality care study

**DOI:** 10.1186/s13244-021-01007-4

**Published:** 2021-05-26

**Authors:** Stefania Rizzo, Maria Del Grande, Vittoria Espeli, Anastasios Stathis, Gabriele Maria Nicolino, Filippo Del Grande

**Affiliations:** 1 Istituto Di Imaging Della Svizzera Italiana (IIMSI), Clinica Di Radiologia EOC, Via Tesserete 46, 6900 Lugano, Switzerland; 2 Facoltà Di Scienze Biomediche, Università della Svizzera italiana (USI), Via G. Buffi 13, 6904 Lugano, Switzerland; 3 Oncology Institute of Southern Switzerland, San Giovanni Hospital, 6500 Bellinzona, Switzerland; 4 Post-Graduate School in Radiodiagnostics, Università Degli Studi Di Milano, Via Festa del Perdono 7, 20122 Milan, Italy

**Keywords:** Radiology report, Radiology subspecialty, Oncologic imaging, Quality

## Abstract

**Background:**

The main objective was to assess whether CT reports of radiologists subspecialized in oncologic imaging respond better to oncological referrals than reports from general radiologists. The secondary objective was to assess differences in ratings between a senior and junior oncologist. Two hundred radiological reports pertaining to oncological patients were retrospectively selected of which 100 each were written by subspecialized radiologists and general radiologists, respectively. The senior and junior oncologists each rated all CT reports using a Likert scale from 1 to 5 (1 = very poor, 5 = excellent) for the following information: anatomical details; interpretation of findings; need for further explanations; appropriateness of conclusions; overall satisfaction. Comparisons between ratings assigned to reports from generalist radiologists and subspecialty radiologists were performed using the Mann–Whitney U test. Agreement between both oncologists was assessed through Gwet's coefficient.

**Results:**

For all but two of the five items obtained from the senior oncologist, oncologists' ratings were significantly higher for subspecialty radiologists' reports (*p* < 0.01); mean values from both oncologists were generally higher for subspecialty reports (*p* < 0.001). Agreement between the senior and junior oncologist in the rating of reports from general and subspecialty radiologists was either moderate to substantial (0.5986–0.6788) or substantial to almost perfect (0.6958–0.8358).

**Conclusions:**

According to a senior and junior oncologist, CT reports performed by subspecialized radiologists in oncologic imaging are clearer, more accurate, and more appropriate in the interpretation and conclusions compared to reports written by general radiologists. Likewise, the overall satisfaction of the oncologist from a subspecialized radiologist report is higher.

**Supplementary Information:**

The online version contains supplementary material available at 10.1186/s13244-021-01007-4.

## Key points

- Radiologists subspecialized in oncologic imaging better report anatomical details and terminology.
- Subspecialized radiologists better interpret imaging findings and draw appropriate conclusions.
- Oncologists prefer reports prepared by subspecialized radiologists than those by general radiologists.

## Background

Traditionally, radiologists have served as general consultants to physicians of various disciplines. They diagnose, indicate treatment decisions, and guide management across many medical diseases, including oncological diseases. With increasing subspecialization in medicine, it is more and more difficult, if not impossible, for general radiologists to keep pace with the rapidly changing knowledge in the different fields of radiology. Such a changing environment increases the risk of losing value in radiology if radiologists are not focusing on selected areas of imaging [1]. Indeed, the American College of Radiology (ACR) encourages radiologists to provide actionable and meaningful reports that add value to the ordering physician, in order to help the transition from volume-based to value-based imaging care [2].

Many articles in the literature have demonstrated the importance of a second interpretation by subspecialty radiologists [3–5]. The subspecialty interpretation is generally sought out to answer specific clinical questions, which may be related to patient symptoms, differential diagnosis, planning of surgical interventions, therapy selection, and prediction of treatment response. For example, in a pilot study of patients presenting with biliary tract disease, non-emergent gastrointestinal bleeding or abdominal mass, the authors reported that diagnostic evaluation made by a consultant subspecialty radiologist resulted in a 64% reduction in time to diagnosis and a 32% reduction in number of studies performed [6]. Chalian et al. reviewed over 2000s-opinion subspecialty consultations in musculoskeletal radiology, and noted clinically relevant different interpretations in 26.2% cases, and discrepant interpretations in 36.3% of oncologic cases [3]. Based on the final pathologic diagnosis, they reported that second-opinion consultations were correct in 82.0% of cases where discrepancies were likely to change patient management [3].

In a similar way, several tertiary oncology care centers have looked at the quantitative effect of radiology consultations on management of cancer patients. One center reported the addition of significant new information in 49% of patients reviewed at their division of oncology radiology meetings, resulting in major management changes in 37% [7]. Two different centers reported a change in interpretation of 41%, with statistically significant changes in tumor node metastases (TNM) staging in 34% of patients, which altered treatment plans in 98% and affected prognosis in 95% [8].

To the best of our knowledge, no previous study has evaluated the value of radiological reports made by radiologists subspecialized in oncologic imaging, for their consistency and meaningfulness according to the clinical indication. Therefore, the main objective of our retrospective study was to assess whether radiological reports of chest and abdomen CT scans performed by radiologists subspecialized in oncologic imaging respond better to the clinical oncological indications than reports written by general radiologists.

The secondary objective was to assess differences in ratings between a senior and junior oncologist.

## Methods

The present study was considered a quality care control study by our Ethics Committee and did not fall under the Swiss law of human research. As such, specific approval and informed consent were waived.

### Radiological report selection

We retrospectively included consecutive reports of chest and abdomen computed tomography (CT) examinations requested by the Oncology Institute of our Hospital network. The radiological reports were retrieved from our radiology information system (RIS) and then divided into two groups: one group consisted of reports by subspecialized radiologists (starting from January 2019 onwards) and one group of reports by general radiologists (from December 2018 backwards). The cutoff of January 2019 was chosen because it corresponded to a re-organization of the Department of Radiology of our Institution, with a subsequent grouping of radiologists according to sub-specialties. Radiologists were considered subspecialized in oncologic imaging and could join the oncologic imaging group if they had at least 5 years of experience in oncologic imaging and if they demonstrated regular participation in educational events dedicated to oncologic imaging. General radiologists were radiologists with 0–20 years of experience, reporting radiological examinations according to modality.

We excluded reports written before December 2018 by radiologists that in 2019 joined the oncologic imaging group later on, and the search went on to find other reports, in order to reach the same number of reports for subspecialized and general radiologists.

### Data records

All the radiological reports in our department are prepared according to the general ACR guidelines [2]. They include the following paragraphs: indication (where the clinical information and clinical question are noted); technical data (including procedure and materials, such as quantity and rate of injection of contrast medium); findings (including description of findings and their interpretation); conclusions (reporting the overall impressions of the examination). The reports were extracted from the RIS, and they were copied and pasted into a separate word file, completely anonymized (not coded) by a radiology resident. First name, second name, date of birth, and gender were canceled from the report. In order to minimize the possibility of recognizing the reporting radiologist, the text format was standardized (i.e., for capital letters in the title, bold font of the headings, etc.) and the technical data were cut because some radiologists use a personal template. The reports selected were initially colored in red and blue to distinguish the ones reported by subspecialists (red) from those reported by generalists (blue), and then mixed, numbered consecutively, and finally colored in black. In a separate Excel spreadsheet file, we recorded the ones from subspecialists and the ones from generalists.

### Clinical rating of the radiological reports

One senior oncologist (V.E.) with 10 years of experience in oncology after board certification and one junior oncologist (M.D.G.) with 3 years of experience in oncology after board certification rated the reports. Based on clinical indication, the two oncologists rated the appropriateness of the following information mentioned in the radiological report: the accuracy of the report, the interpretation of the findings, the clarity of the report, and the appropriateness of the conclusions. Furthermore, also the overall subjective satisfaction of the oncologist was rated.

All the above-mentioned information was rated according to an equidistant Likert scale from 1 to 5, where 1 indicated very poor rating; 2 indicated poor rating; 3 indicated fair rating; 4 indicated good rating; and 5 indicated excellent rating.

For accuracy of the report, the correct use of anatomical details and terminology was considered. For interpretation of findings, the evaluation of imaging finding in the body of the report was considered.

For clarity of the report, the need for further explanations after reading the report was considered.

For appropriateness of conclusions, the appropriate answer to the clinical request was considered. The conclusion had to have a comparison with a previous exam if present, a diagnosis or a differential diagnosis if possible, and a follow-up with the right timing and modality if needed. Furthermore, the readers assigned an overall subjective satisfaction rate, from 1 to 5, where 1 = very unsatisfied; 2 = unsatisfied; 3 = neutral; 4 = satisfied; and 5 = very satisfied.

An overall rating score was determined for each item as the mean of the two ratings reported by each oncologist.

### Statistical analysis

Descriptive statistics were reported as mean ± standard deviation. Comparisons between generalist and subspecialty radiologists’ reports were performed using the Mann–Whitney U test. Interrater agreement was determined using the Gwet's coefficient [9] instead of Cohen's Kappa to avoid the interrater agreement paradox [10]. Levels of agreement were defined using the classification of Landis and Koch as follows: below 0.00 poor; 0.01–0.20 slight; 0.21–0.40 fair; 0.41–0.60 moderate; 0.61–0.80 substantial; and 0.81–1.00 almost perfect [11].

A post hoc power analysis was carried out to check whether nonsignificant results were due to a lack of statistical power (G*Power 3.1 for Macintosh, Heinrich-Heine, Dusseldorf, Germany).

Statistical significance was set at 5% (*p* < 0.05). Analyses were performed using STATA16 for Mac (StataCorp, College Station, TX, USA).

## Results

Two hundred radiological reports were included (100 written by subspecialized radiologists and 100 by generalist radiologists). Sub-specialized radiologists were three; generalist radiologists were > 10.

**Fig. 1:**
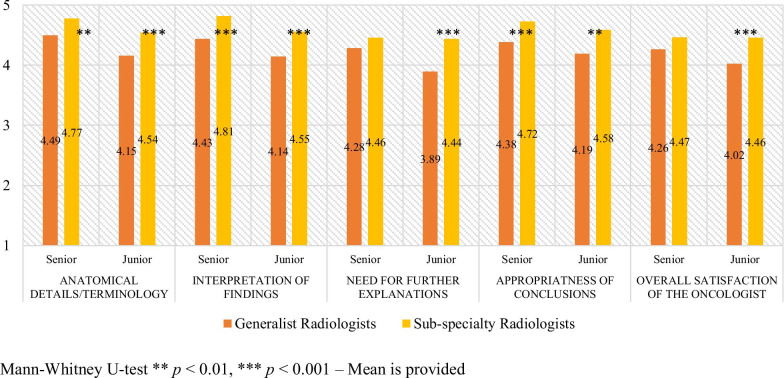
Comparisons between ratings assigned to reports written by generalist radiologists and subspecialty radiologists

As shown in Fig. 1, both clinicians were more satisfied by the subspecialty reports for anatomical details, interpretation of findings, and appropriateness of conclusions. The ratings of the senior oncologist showed no difference between subspecialist/generalist reports in terms of the need for further explanations and for overall satisfaction, whereas for these two information, the junior oncologist was more satisfied by subspecialty radiologists’ reports. A post hoc power analysis showed that the lack of significance recorded regarding two items for the senior oncologist (need for further explanations and overall satisfaction) could be attributed to the limited sample size (see Additional file 1: Table S1).

Table 1 summarizes the mean score and standard deviation for the five variables of the two oncologists and shows that the ratings were significantly higher when the reports were written by subspecialty radiologists for all variables (anatomical details/terminology *p* < 0.0001; interpretation of findings *p* < 0.0001; need for further explanations *p* < 0.001; appropriateness of conclusions *p* < 0.001; overall satisfaction of the oncologist *p* < 0.001).

**Table 1:** Ratings of the 2 oncologists for the reports of the generalist and subspecialty radiologists

	Generalist radiologists	Sub-specialty radiologists	*p* value
Anatomical details/terminology	4.32 ± 0.64	4.66 ± 0.48	< 0.0001
Interpretation of findings	4.28 ± 0.61	4.68 ± 0.45	< 0.0001
Need for further explanations	4.09 ± 0.66	4.45 ± 0.55	< 0.001
Appropriateness of conclusions	4.28 ± 0.60	4.65 ± 0.45	< 0.001
Overall satisfaction of the oncologist	4.14 ± 0.66	4.46 ± 0.52	< 0.001

Table 2 shows that an almost perfect agreement between the clinicians emerged on subspecialty reports regarding anatomical details/terminology (AC = 0.8358) and the appropriateness of conclusions (AC = 0.8216). Substantial agreement was achieved on subspecialty reports about interpretation of findings (AC = 0.6958), need for further explanation (AC = 0.7012), and overall satisfaction of the report (AC = 0.7078). Moderate and substantial levels of agreement were observed on generalist radiologists’ reports about the need for further explanations (AC = 0.5986), anatomical details/terminology (AC = 0.6788), interpretation of findings (AC = 0.6762), appropriateness of conclusions (AC = 0.6731), and overall satisfaction of the report (AC = 0.6647).

**Table 2:** Agreement between senior and junior oncologists (Gwet’s coefficient)

		Junior oncologist	
		Generalist radiologists	Sub-specialty radiologists
Anatomical details/terminology	Senior oncologist	0.6788	0.8358
Interpretation of findings	Senior oncologist	0.6762	0.6958
Need for further explanation	Senior oncologist	0.5986	0.7012
Appropriateness of conclusions	Senior oncologist	0.6731	0.8216
Overall satisfaction of the oncologist	Senior oncologist	0.6647	0.7078

Level of agreement: below 0.00 poor; 0.01–0.20 slight; 0.21–0.40 fair; 0.41–0.60 moderate; 0.61–0.80 substantial; 0.81–1.00 almost perfect

## Discussion

In this study, we have demonstrated that, according to the oncologists, reports prepared by subspecialized radiologists in oncologic imaging responded better to clinical indications for anatomical details and interpretation of findings, needed fewer explanations, and were more appropriate in their conclusions than reports written by generalist radiologists. Moreover, the overall subjective satisfaction of the oncologist was significantly higher for the reports written by subspecialized radiologists, and the agreement between oncologists increased when the reports were by subspecialty radiologists compared to general radiologists.

Meaningful radiology reports are essential to help referring physicians in their decision-making process and to increase the quality of care for patients.

Unfortunately, there is a lack of a standard method to evaluate the quality radiology reports. On the one hand, many previous studies have underlined the importance of imaging interpretation, by assessing the impact on clinical decisions or changes in treatment after a second opinion given by subspecialized radiologists [12–14]. On the other hand, it has been stated that the radiology report must not only be accurate but also meaningful [15, 16]. Indeed, according to a Canadian survey, clarity and meaningfulness were the most valued qualities of radiology reports among 200 referring physicians [17]. In the context of radiology reporting, the radiologist's message may not be accurately conveyed to the referring physician, and at other times, the referring physician may misunderstand the radiologist’s report [18].

In our study, both oncologists found that the anatomical details were significantly better explained, and the terminology was more accurate, in the reports made by subspecialized radiologists compared to those made by generalist radiologists. Anatomical details and correct terminology are important facts in oncologic patients at staging, in order to evaluate the possibility of surgical excision, as well as at follow-up, when infiltration of adjacent structures may explain symptoms and may be considered at risk for complications and needing further treatments [19, 20]. This kind of information is frequently dealt with by radiologists in multidisciplinary meetings, where specific questions are posed to decide the most appropriate treatment path [4, 5]. Typical anatomical details needed for oncological patients, for instance, may be the site of retroperitoneal lymph nodes related to the renal vessels for patients with gynecological malignancy who are candidates for retroperitoneal lymphadenectomy, or the relationship between tumoral tissue and mesenteric vessels for pancreatic cancer, or between the malignant tissue and the main pulmonary artery for lung cancer. Our study demonstrates that some of this information may already be mentioned in the original report.

According to both oncologists, imaging findings were significantly better interpreted by subspecialized radiologists than by general radiologists. Indeed, while for a radiologist the description of a hepatic lesion based on the CT density can be evident, this is not the case for the referring oncologist, who needs to know if it is a benign or malignant lesion and, when malignant, if it is more likely a primary or a secondary lesion. Likewise, the mention of the presence of a lymph node should be complemented by its interpretation as positive or negative [21], and the description of a ground-glass opacity should be accompanied by a level of suspiciousness for malignancy [22].

A significant difference in the need for further explanations was recorded only for the junior oncologist. We can speculate that, according to his longer experience, the senior oncologist needs less help through explanations from the radiologists, compared to the junior oncologist. These results are even more interesting because they indirectly indicate that when the imaging report is written by a subspecialized radiologist, the number of phone calls to the radiology department can be lowered. Consequently, as demonstrated by Zabel et al. [23], we may hypothesize that along with a higher value of the radiological report, another advantage of the subspecialty-based organization is a more efficient workflow. This can be important in centers recruiting patients in clinical trials, where a dedicated radiologist is sometimes requested. If the reports are always written by dedicated radiologists, a second reading could be avoided.

The conclusions represent the final impressions and are one of the most challenging parts of the report. According to the ACR guidelines, the conclusions should contain a specific diagnosis, or a differential diagnosis when appropriate, and suggestions for follow-up or additional diagnostic studies to clarify or confirm the impression [2]. In our series, both the oncologists indicated that the conclusions reported by the subspecialized radiologists were significantly more appropriate than those made by generalists.

Of note, with the exception of the variable “need for further explanations” rated by the junior oncologist with a mean of 3.89, the mean ratings of both oncologists are all above 4 on the scale from 1 to 5. Likewise, the senior oncologist did not find a statistically significant difference in the overall satisfaction provided by the reports regardless who has written the report. This indicates that the quality and usefulness of the reports provided by general radiologists were already fairly high. However, the process of upgrading the quality and meaningfulness of radiological reports should aim to the best, not to the fairly high. Furthermore, not all the oncological cases are discussed in multidisciplinary meetings, some oncologists for example do private practice; therefore, the highest quality and usefulness of the radiological reports by subspecialized radiologists might help the decision making process also outside a dedicated oncological hospital.

Worthy of mention is the fact that the inter-reader reliability for all five variables was higher for the radiological reports written by subspecialty radiologists compared to those written by general radiologists.

This study has some limitations. First, the rating of the reports was made without reviewing the images, and therefore, the accuracy of the content of the report was not evaluated. However, this topic has been evaluated elsewhere in the literature and was beyond the scope of this study. Second, the subspecialized radiologists did not have specific board certifications because oncologic imaging does not belong to the subspecialties currently requiring additional training and examination. Nevertheless, they joined the oncologic imaging group based on their own experience and their participation in specific educational events. Third, we did not perform a preliminary power analysis to assess the appropriate number of reports to include. However, we performed a post hoc analysis demonstrating that the results that were not significant (*p* > 0.05) were related to the limited sample size, and this is clearly stated in the results. Finally, rating of the radiology reports was based on the clinical indication mentioned at the beginning of the report, and we are aware that the clinical history given to the radiologist can sometimes be lacking in detail. However, we did not investigate this in more depth, in order to reproduce a real-life situation as much as possible, where the radiologist looks at the images and provides responses to what is required as a clinical indication.

In conclusion, our study demonstrated that radiology reports of chest and abdomen CT scans written by subspecialized radiologists in oncologic imaging respond better to the clinical indication compared to those written by general radiologists, and that the agreement between oncologists increased when the reports are prepared by subspecialty radiologists. Radiology reports written by subspecialized radiologists in oncology imaging are indeed clearer, more accurate, more appropriate in their conclusions and interpretation, and more reliable compared to radiology reports written by general radiologists.

## Supplementary Information

**Additional file 1**: Table S1. Effect size and power of the post hoc analysis.

## Data Availability

Not applicable.

## Abbreviations

ACR: American congress of radiology
CT: Computed tomography
RIS: Radiology information system
TNM: Tumor node metastases
