# Supplementary material for: Do oncologists prefer subspecialty radiology reports? A quality care study
Source: Insights Imaging. 2021 May 26;12:64. doi: 10.1186/s13244-021-01007-4 (PMC8155173; doi:10.1186/s13244-021-01007-4)
Supplement: Supplementary file 1 — Additional file 1: Table S1. Effect size and power of the post hoc analysis. [file 13244_2021_1007_MOESM1_ESM.docx]

**ELECTRONIC SUPPLEMENTARY MATERIAL**

**Supplementary Table 1.** Effect size and power of the post-hoc analysis

|  |  | Effect Size | Power |
| --- | --- | --- | --- |
| **Anatomical details/terminology** | **Senior** | 0.44 | 0.91 |
|  | **Junior** | 0.50 | 0.96 |
| **Interpretation of findings** | **Senior** | 0.59 | 0.99 |
|  | **Junior** | 0.57 | 0.99 |
| **Need for further explanations** | **Senior** | 0.24 | 0.49 |
|  | **Junior** | 0.68 | 0.99 |
| **Appropriateness of conclusions** | **Senior** | 0.50 | 0.96 |
|  | **Junior** | 0.54 | 0.98 |
| **Overall satisfaction of the oncologist** | **Senior** | 0.29 | 0.63 |
|  | **Junior** | 0.55 | 0.98 |
